# Awareness and Knowledge of Pre-eclampsia Among Saudi Women of Reproductive Age

**DOI:** 10.7759/cureus.49233

**Published:** 2023-11-22

**Authors:** Ashraf Radwan, Manar Al Naji, Nourah Alyoubi, Iram Alsallat, Zakeiah Alsulaimani, Shaima Ali Albeladi, Hussein Sabban, Abdulmageed Abdou, Ali Alsamry

**Affiliations:** 1 Obstetrics and Gynaecology, King Abdulaziz University Faculty of Medicine, Jeddah, SAU; 2 Medicine, King Abdulaziz University Faculty of Medicine, Jeddah, SAU; 3 Medicine and Surgery, King Abdulaziz University Faculty of Medicine, Jeddah, SAU

**Keywords:** pregnancy, hypertensive, pre-eclampsia, knowledge, awareness

## Abstract

Background: Pre-eclampsia has emerged as a significant concern in maternal healthcare worldwide, impacting the health and well-being of expectant mothers and their unborn children. This study examines the extent of pre-eclampsia knowledge and awareness among Saudi women aged 18 to 49. Recognising informed and proactive healthcare decisions is pivotal in managing and preventing pre-eclampsia.

Methods: It is a prospective cross-sectional community-based study design. We calculated a minimum sample size of 385 participants using the Raosoft online calculator, aiming for a 5% margin of error and a 95% confidence interval. The structured questionnaire was distributed via various social media platforms to collect the data. The questionnaire consisted of two sections, namely demographics and medical history. Additionally, the questionnaire explored pre-eclampsia risk factors, symptoms, and complications. We analysed data using the Statistical Package for the Social Sciences (SPSS) version 26 (IBM Corp., Armonk, NY). We applied statistical tests, including the Chi-squared test (χ2) and the Mann-Whitney test for non-parametric variables.

Results: Most of our participants were young, with a mean age of 25.94 and single (69.5%). Moreover, many (68.8%) had a bachelor's degree. A notable portion of participants stated they had no family history (86.1%) of pre-eclampsia and (98.1%) no previous experience with pre-eclampsia. Approximately 50% recognised hypertension as a symptom, while 44.1% identified persistent headaches. However, complications such as fetal and maternal death were better-known consequences of pre-eclampsia. 11.3% of participants had adequate knowledge about pre-eclampsia, 39.3% had moderate knowledge, and 49.4% had inadequate knowledge. It is a fact that higher levels of knowledge are positively correlated with advanced age, prior pregnancy experience, and a family history of pre-eclampsia.

Conclusion: This study highlights the limited knowledge and awareness of pre-eclampsia among Saudi women of reproductive age. Addressing this knowledge gap is crucial for preventing pre-eclampsia-related maternal and fetal complications. Policymakers and healthcare providers should consider implementing educational programs to raise awareness and improve outcomes for pregnant women in Saudi Arabia and similar regions.

## Introduction

Pre-eclampsia is a hypertensive pregnancy disorder that usually occurs after 20 weeks [1], and It is characterised by new-onset hypertension, usually after 20 weeks of gestational proteinuria, frequently accompanied by new-onset proteinuria.

The severity of maternal hypertension, the presence or absence of proteinuria, and the presence or absence of other clinical signs of the disease are all highly variable. When hypertension and proteinuria are detected, some women may be asymptomatic, whereas others may exhibit severe pre-eclampsia symptoms such as visual abnormalities, severe headaches, or upper abdominal pain [2].

Pre-eclampsia affects 2% to 8% of pregnant women and is a significant cause of maternal and perinatal morbidity and mortality [3-5]. Pre-eclampsia affects approximately 5.37 of every 10,000 women in Saudi Arabia [6].

Pregnancy-induced hypertension is responsible for 10% of global maternal deaths, with 30,000 women dying yearly due to hypertension caused by pregnancy [7]. Although maternal death from pre-eclampsia is less common in developed countries, maternal morbidity is high and contributes significantly to ICU admissions during pregnancy [8,9].

Pre-eclampsia causes 12 to 25% of fetal growth restriction, small-for-gestational-age infants, and 15% to 20% of all preterm births. Prematurity has many serious consequences, including neonatal deaths and long-term neonatal morbidity [8-10]. Three systematic reviews identified previous pre-eclampsia, maternal age >40, pregnancy BMI >30, and prior stillbirth as risk factors for pre-eclampsia. Nulliparity, chronic hypertension, multiple fetal births, increased pre-pregnancy BMI, long inter-pregnancy interval (> five years), chronic renal disease, gestational diabetes, placental abruption, assisted reproduction, and prior intrauterine growth restriction, systemic lupus erythematosus, and antiphospholipid antibody syndrome [11-13]. The cause of pre-eclampsia is unknown. Current theories suggest that genetic factors, placental ischemia, immunological maladaptation, and vascular mediators may all play a role in its development [14].

The various study objectives, sample types (for example, gender, prior experience with pre-eclampsia, being a "patient" or a "relative"), sample sizes, and different items reflect the outcomes of other studies. The research was conducted across various socio-cultural contexts. Regardless of population or location, most of this published work suggests that pre-eclampsia knowledge levels are frequently low [15-20].

Pre-eclampsia awareness, early diagnosis, and comprehensive care throughout pregnancy can all help prevent maternal and fetal complications. This study aims to assess pre-eclampsia understanding among Saudi women of reproductive age.

Preventing maternal and fetal complications can be accomplished through public education, pre-eclampsia awareness, early diagnosis, and comprehensive care throughout pregnancy.

## Materials and methods

Study design, setting and time

A cross-sectional study was conducted in Saudi Arabia between June and August 2023.

Study participants

The inclusion criteria were Saudi women with an age ranging from 18-49 years. Moreover, the exclusion criteria were non-Saudi women and those under 18 or above 49 years old.

Sample size

Based on the calculations performed by the Raosoft online calculator website, a minimum sample size of 385 participants with a 5% margin of error and a 95% confidence interval is required. We recruited 1169 consecutive consenting pregnant women to increase the study's statistical power.

Data collection

A self-constructed Google Form questionnaire was created based on existing literature, and experts were consulted to ensure its validity in the context of public health [21]. A questionnaire was distributed randomly across social media platforms such as WhatsApp, Telegram, and Instagram. To ensure inclusivity, we also made an Arabic version of the questionnaire so that people from diverse backgrounds could participate (see Appendices).

The questionnaire has been distinctly separated into two crucial sections. The initial part comprises socio-demographic factors such as age, gender, educational background, marital status, employment status, and place of residence. It also includes a medical history of pre-eclampsia.

The second section asked the participants about their pre-eclampsia knowledge and experience. First, it focused on whether they had known about pre-eclampsia, if they had experienced it themselves, and if they had a family history of pre-eclampsia. Then, the questionnaire focused on pre-eclampsia and its signs and symptoms. Participants were asked to confidently indicate whether high blood pressure, persistent headaches, swelling, blurred vision, chest pain, abdominal pain, nausea and vomiting, and back pain were signs or symptoms of pre-eclampsia. In addition, the questionnaire addressed the risk factors associated with pre-eclampsia, such as having a family history of the condition, obesity, diabetes, unhealthy lifestyle, and multiple births.

The questionnaire also explored the complications associated with pre-eclampsia. Participants were asked to confidently indicate whether maternal death, fetal death, heart disease, and kidney dysfunction are complications of pre-eclampsia. Furthermore, the questionnaire asked whether pre-eclampsia is more likely to occur before 20 weeks of gestation or after 20 weeks of gestation. Participants were also asked to indicate their knowledge of the severity of pre-eclampsia as very severe, severe, not severe, or unsure. Finally, the participants were asked if they were worried about pre-eclampsia or not.

Using a scoring system, we evaluated the level of knowledge and awareness among females regarding pre-eclampsia in section two. This information is crucial in understanding the gaps in current levels of awareness and can help us devise strategies to improve it. The scoring system assigned one point for each "Yes" answer and zero for any "No" or "I do not know" answers. The knowledge score ranged from 0 to 22, depending on the participant's knowledge level. The participant's knowledge level needs to be improved if the score is between 0-11. Scoring between 11-16 means that the knowledge level is moderate while scoring between 16-22 indicates an adequate knowledge level.

Ethical considerations

The ethical approval obtained from the research ethics committee of King Abdulaziz University (KAU) in Jeddah, Saudi Arabia (Reference No. 426-23) proves our commitment to conducting a study that adheres to the highest ethical standards. Before participating in the study, participants were required to provide informed consent, and the study's primary objective was clearly explained to them. Also, they are informed that participation in this research is optional and does not entail any benefits, and the information given will remain confidential.

Data analysis

Data were analysed using the Statistical Package for the Social Sciences (SPSS) version 26 (IBM Corp., Armonk, NY). To assess the association between the variables, the Chi-squared test (χ2) and the Fisher's Exact Test were used for qualitative data that was expressed as numbers and percentages. Quantitative data was presented as mean and standard deviation (Mean ± SD), where the Mann-Whitney test was applied for non-parametric variables. A P-value of <0.05 was considered as statistically significant.

## Results

**Figure 1:**
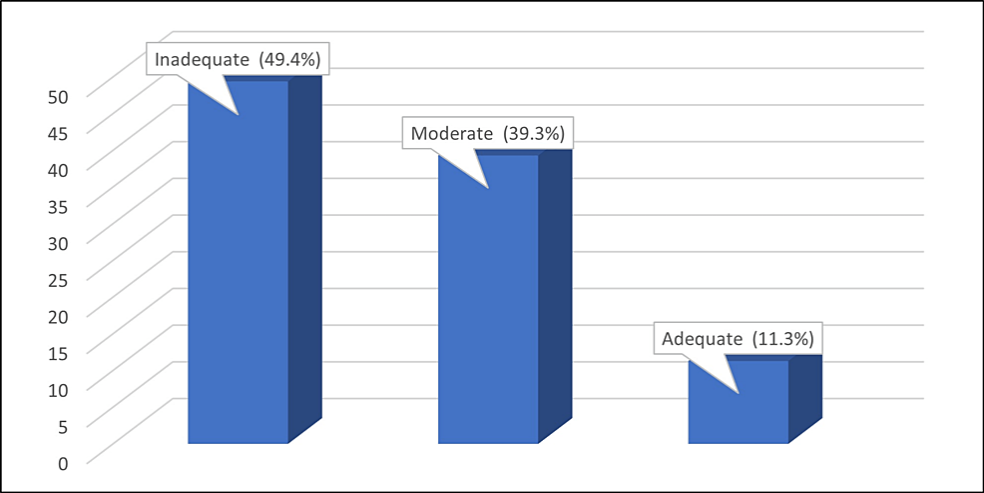
Percentage distribution of the level of knowledge about pre-eclampsia among studied participants Number of studied participants: 1169

The study analysed 1,169 participants to determine the level of awareness regarding pre-eclampsia. The study's results indicate that a significant proportion of participants, approximately 49.4%, had inadequate knowledge, while 39.3% had moderate knowledge. Only 11.3% of the participants displayed adequate knowledge. The outcomes of the study are presented in Figure 1.

Table 1* *provides an overview of the demographic characteristics of the study participants, including their pregnancy status and pre-existing history of pre-eclampsia, either in their family or themselves. The study encompassed 1169 participants. Based on the study results, the mean age of the participants was 25.94 years, with a standard deviation of 7.94 years.

**Table 1:** Distribution of studied participants according to their demographic characters, pregnancy, and previous experience of pre-eclampsia in oneself or a family member Number of studied participants: 1169

Variable	No. (%)
Age (years)	25.94 ± 7.94
Marital status	
Divorced	31 (2.7)
Married	317 (27.1)
Single	813 (69.5)
Widow	8 (0.7)
Level of education	
Elementary school	3 (0.3)
Middle school	6 (0.5)
High school	302 (25.8)
Bachelor degree	804 (68.8)
Higher education	54 (4.6)
Occupation	
Unemployed	900 (77)
Employed	269 (23)
Are you pregnant or have been pregnant	
Does not apply	124 (10.6)
No	758 (64.8)
Yes	287 (24.6)
Have you experienced pre-eclampsia before	
No	1147 (98.1)
Yes	22 (1.9)
Do you have a family history of pre-eclampsia	
No	1006 (86.1)
Yes	163 (13.9)

Regarding marital status, most participants were single, accounting for 69.5% of the sample, while 27.1% were married. Interestingly, only a negligible proportion of participants reported being divorced (2.7%) or widowed (0.7%). Concerning educational attainment, a significant percentage of participants had completed a bachelor's degree (68.8%), while 25.8% had a high school education. However, a relatively small percentage of participants had higher education (4.6%), attended middle school (0.5%), or completed elementary school (0.3%).

Furthermore, most participants (77%) were unemployed, while 23% were employed. Concerning pregnancy status, 64.8% of participants were not pregnant, 24.6% were pregnant, and 10.6% indicated that the question was not applicable. It is important to note that most participants (98.1%) had no prior experience with pre-eclampsia, while only a small proportion (1.9%) had previous exposure to the condition. Regarding pre-eclampsia, 86.1% of participants reported no family history, while 13.9% reported having a family history.

**Figure 2:**
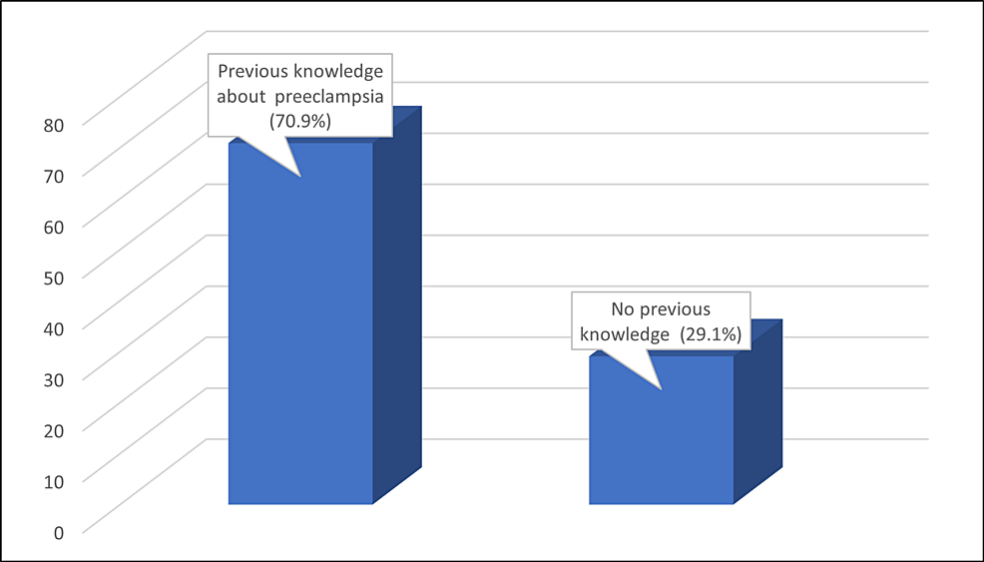
It is worth noting that the data presented in this figure shows that the majority of participants (70.9%) had prior knowledge of pre-eclampsia before participating in the study

It is worth noting that the data presented in Figure 2 shows that the majority of participants (70.9%) had prior knowledge of pre-eclampsia before participating in the study.

The participant's responses to the knowledge survey on pre-eclampsia risk factors, timing, complications, and severity have been documented in Table 2. The results showed that half of the participants (50%) agreed that hypertension during pregnancy is one of the signs/symptoms of pre-eclampsia. The frequency of agreement to other pre-eclampsia symptoms was as follows: persistent headache (44.1%), oedema (49.1%), blurred vision (39.3%), chest pain (25.1%), abdominal pain (49.4%), nausea and vomiting (57.8%), and back pain (38.8%).

**Table 2:** Participants' responses to knowledge items regarding risk factors, complications, timing and severity of pre-eclampsia Number of studied participants: 1169

Variable	I don't know No. (%)	No No. (%)	Yes No. (%)
Knowledge about signs/symptoms of pre-eclampsia			
High blood pressure (during pregnancy)	408 (34.9)	176 (15.1)	585 (50)
Persistent headache	469 (40.1)	185 (15.8)	515 (44.1)
Oedema	423 (36.2)	172 (14.7)	574 (49.1)
Blurred vision	498 (42.6)	211 (18)	460 (39.3)
Chest pain	610 (52.2)	265 (22.7)	294 (25.1)
Abdominal pain	413 (35.3)	178 (15.2)	578 (49.4)
Nausea and vomiting	343 (29.3)	150 (12.8)	676 (57.8)
Back pain	539 (46.1)	176 (15.1)	454 (38.8)
Knowledge about risk factors for pre-eclampsia			
Family history of pre-eclampsia	428 (36.6)	422 (36.1)	319 (27.3)
Having prior pre-eclampsia	343 (29.3)	121 (10.4)	705 (60.3)
Obesity	377 (32.2)	114 (9.8)	678 (58)
Diabetes	332 (28.4)	8.6 (7.4)	751 (64.2)
Unhealthy lifestyle	268 (22.9)	83 (7.1)	818 (70)
Multiple births	577 (49.4)	368 (31.5)	224 (19.2)
Knowledge about complications of pre-eclampsia			
Maternal death	440 (37.6)	149 (12.7)	580 (49.6)
Fetal death	297 (25.4)	68 (5.8)	804 (68.8)
Heart diseases	736 (63)	253 (21.6)	180 (15.4)
Kidney dysfunction	637 (54.5)	154 (13.2)	378 (32.3)
When is one likely to experience pre-eclampsia?			
< 20 weeks of gestation	361 (30.9)		
≥ 20 weeks of gestation	808 (69.1)		
How severe is pre-eclampsia?			
I don’t know	616 (52.7)		
Not severe	36 (3.1)		
Severe	252 (21.6)		
Very severe	265 (22.7)		

Regarding pre-eclampsia risk factors, 27.3% agreed that a family history of pre-eclampsia is a risk factor, while 60.3% agreed that having prior pre-eclampsia is a risk factor. The frequency of agreement on other pre-eclampsia risk factors was as follows: obesity (58%), diabetes (64.2%), unhealthy lifestyle (70%), and multiple births (19.2%).

We asked the participants to report the most common complications associated with pre-eclampsia. The results showed that fetal death was the most frequently reported complication, with 68.8% of participants indicating it as a concern. Maternal death was also a significant concern, with 49.6% of participants citing it as a potential complication. Kidney dysfunction (32.3%) and heart disease (15.4%) were other commonly reported complications. Most studied females (69.1%) reported that pre-eclampsia will likely occur after 20 weeks of gestation. Moreover, 22.7% considered pre-eclampsia very severe, while only 21.6% reported it mild.

According toFigure 3, it is encouraging to note that half of the participants (50.3%) expressed caution about pre-eclampsia, which is a positive outcome. Furthermore, the mean knowledge score of 11.31 ± 5.05 indicates that the participants had a satisfactory level of understanding of the condition.

**Figure 3:**
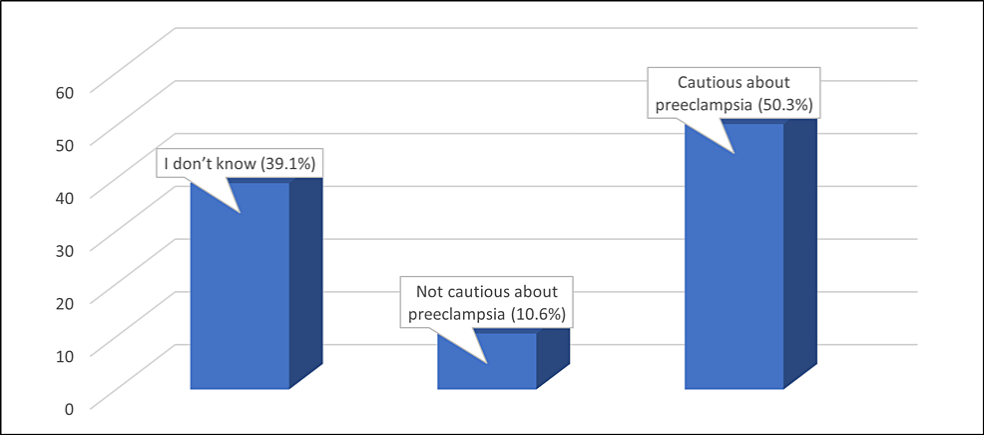
Percentage distribution of how the participants are cautious about pre-eclampsia Number of studied participants: 1169

Table 3 demonstrates a clear association between pre-eclampsia knowledge level and participants' demographics, pregnancy status, and family history. We categorised participants into three groups based on their knowledge level: inadequate, moderate, and adequate.

**Table 3:** Relationship between knowledge level about pre-eclampsia and participants' demographics, pregnancy, and family history of pre-eclampsia * = Fisher's Exact Test Number of studied participants: 1169

Variable	Knowledge level			χ2	p-value
	Inadequate No. (%)	Moderate No. (%)	Adequate No. (%)		
Age	25.59 ± 7.34	27.13 ± 8.77	25.46 ± 6.92	17.49	<0.001
Marital status					
Divorced	11 (1.9)	16 (3.5)	4 (3)	12.24*	0.051
Married	143 (24.7)	136 (29.6)	38 (28.8)		
Single	423 (73.2)	301 (65.6)	89 (67.4)		
Widow	1 (0.2)	6 (1.3)	1 (0.8)		
Level of education					
Elementary school	1 (0.2)	2 (0.4)	0 (0.0)	13.3*	0.068
Middle school	4 (0.7)	0 (0.0)	2 (1.5)		
High school	151 (26.1)	121 (26.4)	30 (22.7)		
Bachelor degree	404 (69.9)	307 (66.9)	93 (70.5)		
Higher education	18 (3.1)	29 (6.3)	7 (5.3)		
Occupation					
Unemployed	454 (78.5)	342 (74.5)	104 (78.8)	2.62	0.269
Employed	124 (21.5)	117 (25.5)	28 (21.2)		
Are you pregnant or have been pregnant					
Does not apply	69 (11.9)	42 (9.2)	13 (9.8)	19.04	0.001
No	398 (68.9)	276 (60.1)	84 (63.6)		
Yes	111 (19.2)	141 (30.7)	35 (26.5)		
Do you have a family history of pre-eclampsia					
No	529 (91.5)	376 (81.9)	101 (76.5)	30.96	<0.001
Yes	49 (85)	83 (18.1)	31 (23.5)		

The results indicate a significant correlation between age and pre-eclampsia knowledge level, with older participants having a higher level of knowledge (χ2 = 17.49, p < 0.001). However, there was no significant correlation between knowledge level and marital status (χ2 = 11.95, p = 0.063) or level of education (χ2 = 13.61, p = 0.106). Similarly, there was no significant relationship between knowledge level and occupation (χ2 = 2.62, p = 0.269).

Regarding pregnancy status, there was a significant correlation between knowledge level and whether the participants were pregnant or had been pregnant (χ2 = 19.04, p = 0.001). Participants who were pregnant or had been pregnant had a higher level of knowledge about pre-eclampsia than those who were not.

Furthermore, there was a significant correlation between knowledge level and family history of pre-eclampsia (χ2 = 30.96, p < 0.001). Participants with a family history of pre-eclampsia had a higher level of knowledge about pre-eclampsia compared to those who did not have a family history

**Figure 4:**
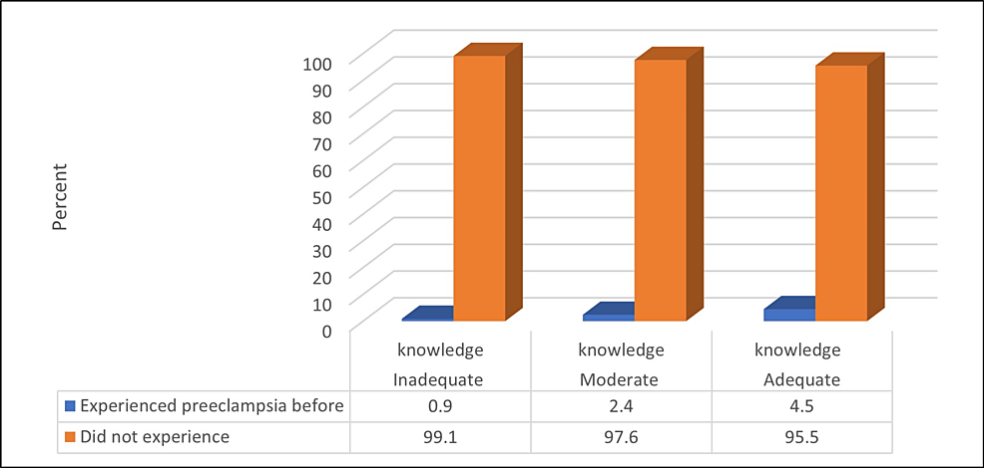
Relationship between knowledge level about pre-eclampsia and previous experience of pre-eclampsia Number of studied participants: 1169

Understanding the connection between knowledge of Preeclampsia and prior experience is crucial, as demonstrated in Figure 4. The results indicate a significant relationship between the knowledge level and the previous experience of pre-eclampsia (χ2 = 8.96, p = 0.011). Participants who had prior experience of pre-eclampsia had a greater understanding of pre-eclampsia compared to those who did not have any previous experience.

In summary, the results suggest that age, pregnancy status, family history, and previous experience of pre-eclampsia are significant factors associated with knowledge level about pre-eclampsia. These findings highlight the need for targeted education and awareness-raising programs for younger women, those without a family history of pre-eclampsia, and those who have not experienced pre-eclampsia in their previous pregnancies.

## Discussion

This cross-sectional study conducted in Saudi Arabia from June to August 2023 aimed to assess the knowledge and awareness of pre-eclampsia among Saudi women aged 18-49. Our findings provide valuable insights into the current knowledge regarding this serious pregnancy-related condition and highlight the factors influencing awareness.

Our study's demographic profile revealed that most participants were young, with a mean age of 25.94 years. Notably, a significant proportion of participants were single (69.5%), which aligns with similar findings in a study conducted by Ejike et al. in Tanzania [22]. In contrast, studies by Fondjo et al. in Ghana [21], Teng et al. in Malaysia [23] and Joseph et al. in India [24] showed different demographic patterns related to the smallest number of participants compared to our study. Our study participants (68.8%) had completed a bachelor's degree, while (77%) were unemployed. The number of employed participants in Tanzania [22], Malaysia [23], Ghana [21] and India [18] was higher than in our study. These variations underscore the diversity within our sample and the potential impact of participant characteristics on the generalizability of our findings.

An intriguing result is the influence of age on knowledge levels, with older participants demonstrating higher knowledge levels (χ2 = 17.49, p < 0.001). This observation aligns with studies conducted in Ghana, where the P value = 0.049 [21] and Malaysia's P value = 0.046 [23], suggesting that increased life experience and exposure to various situations and information may contribute to greater awareness of pre-eclampsia. This underscores the need to target educational efforts for younger women to enhance their understanding of this condition.

Surprisingly, our study found no significant correlation between knowledge and level of education. In contrast to findings from studies in Ghana, it was significant P= 0.008 [21]. Also, Ethiopian research revealed knowledge of preeclampsia was found to be low among women with no education (AOR = 0.22, 95 % CI (0.06, 0.85) [25]. This discrepancy may be attributed to the homogeneity in the level of education among our participants. It is worth noting that educational level alone may not be the sole determinant of knowledge; other factors, such as access to healthcare information and experiences, may play a significant role.

A significant finding was that the majority of participants (98.1%) had no prior experience with pre-eclampsia, and 86.1% reported no family history, which is consistent with studies conducted in Ghana [21] and Ethiopia [25]. This observation is significant since pre-eclampsia has a known genetic component, and family history is a considerable risk factor. The fact that 86.1% of participants reported no family history underscores the absence of a known genetic predisposition in a substantial portion of the study population. Notably, there was a significant correlation between knowledge levels and family history of pre-eclampsia (χ2 = 30.96, p < 0.001), indicating that participants with a family history tended to possess higher knowledge levels.

Our study demonstrates a higher awareness of pre-eclampsia signs and symptoms, with 50% recognising hypertension during pregnancy and 44.1% identifying persistent headaches compared to 39.6% and 31.9% in Ghana [21]. The two studies also show disparity in awareness levels regarding risk factors and complications of pre-eclampsia. In our study, 27.3% of participants recognised a family history of pre-eclampsia as a risk factor, while 60.3% identified a prior pre-eclampsia as a risk factor. Conversely, in a study done in Ghana [21], a higher proportion of participants acknowledged a family history of pre-eclampsia. (37.6%), but fewer recognised prior pre-eclampsia (33.3%) as a risk factor.

Similarly, the order of recognition for complications varied, with maternal death (47.9%) and fetal death (45.6%) being the most accurately reported in our study, while fetal death (68.8%) was the most frequently reported in Ghana [21], followed by maternal death (49.6%). These discrepancies underscore potential differences in participant demographics, healthcare access, and regional influences, which should be considered when interpreting and addressing knowledge gaps.

A critical aspect of our study was assessing participants' knowledge of pre-eclampsia. Notably, a significant proportion of participants (70.9%) were already familiar with pre-eclampsia before participating in the study. This baseline awareness could be attributed to various factors, including prior exposure to healthcare information, personal experiences, or social networks. Understanding the sources of this existing knowledge is crucial for tailoring effective educational efforts, and this finding is similar to a study conducted in Ghana [21] but contrasts with one in Malaysia [23]. Notably, there was a significant correlation between knowledge levels and previous experiences of pre-eclampsia (χ2 = 8.96, p = 0.011). This suggests that individuals who had personal experiences or were close to someone who had experienced pre-eclampsia were more likely to possess higher levels of knowledge.

Our study revealed that most participants (49.4%) had inadequate knowledge, while a substantial portion (39.3%) possessed only moderate knowledge about pre-eclampsia. Alarmingly, only a small percentage (11.3%) exhibited adequate knowledge. This distribution is similar to findings in studies conducted in the USA [15], Malaysia [23], Ghana [21], Tanzania [22], Ethiopia [25], India [18] and Tanzania [26]. However, this contrasts with a study in India [24], where most participants had moderate knowledge.

José and Kharde found that the level of knowledge revealed the majority of mothers, 80(74.07%), had average knowledge, 14(12.96%) had good and poor knowledge; and with regards to self-care measures (60.18%) had average knowledge, 41(37.96%) had good knowledge and 2(1.85%) had poor knowledge, association between the knowledge of mothers and selected variables shows an association with knowledge scores at 0.05 level of significance [19]. They refer their result to the availability of proper antenatal care that is competent and accessible, as well as educational programs for women of reproductive age.

The reason for the lack of awareness about pre-eclampsia may be due to insufficient personal experience, inadequate health companions, and insufficient coverage on social media. However, this study has shown that this issue is not restricted to a particular region but rather a global one. This perspective benefits researchers and policymakers who aim to address the awareness of pre-eclampsia on a broader scale.

Limitations

It is essential to acknowledge that this study has some limitations. Firstly, the sample selected for this study does not represent the general population in Saudi Arabia. Additionally, the data collected in the study was self-reported by women, which means that factors such as recall or social desirability bias could have influenced the results. As a result, some participants may have either over or under-reported their awareness and knowledge of pre-eclampsia, which could skew the study's findings.

It is important to note that the study's questionnaire only captured participants' awareness and knowledge at a specific time without accounting for any changes or improvements that may occur over time.

Furthermore, the study focused on Saudi women, meaning the findings may not apply to women in other cultural contexts or countries. This limitation affects the study's external validity.

Recommendations

Raising awareness about pre-eclampsia is crucial. To achieve this, an educational program should be developed to improve women's understanding of the condition. The program should be user-friendly, interactive, and easily accessible to women of reproductive age.

One effective approach is to use social media and community events to disseminate information and raise awareness about the risks and symptoms of pre-eclampsia. We can educate more women about this potentially life-threatening condition by contacting diverse population segments.

It is also essential to work with community leaders, influential individuals, and women's associations to promote awareness and empower women to take charge of their health by seeking appropriate medical care, including prenatal services.

Follow-up studies should be conducted to assess the effectiveness of these interventions and identify any gaps that need to be addressed in future awareness campaigns.

Improving patient knowledge of pre-eclampsia has been shown to enhance earlier reporting of signs and symptoms, leading to timely care and better health outcomes for both the mother and baby. This is crucial in reducing the prevalence, complications, and mortalities associated with the disease.

## Conclusions

Our study provides a comprehensive assessment of pre-eclampsia knowledge among Saudi women, highlighting areas where education and intervention can make a significant impact. By identifying gaps in awareness, particularly among younger women, we can develop targeted programs to improve understanding of this condition. Moreover, our findings point to preventive strategies that can help reduce the risk of pre-eclampsia, which can lead to better health outcomes for mothers. Overall, this study can inform healthcare policies and interventions in Saudi Arabia and beyond, ultimately contributing to improved maternal health. beyond, leading to improved maternal health outcomes.

## Appendices

You can access the link to the "Knowledge and awareness of pre-eclampsia among reproductive-age women in Saudi Arabia" questionnaire at:

https://docs.google.com/forms/d/e/1FAIpQLSdl2D4PGypLwBYa26jJnOosRTT0yo2kC_gSnHp3kQGspp3QKQ/viewform
